# South Carolina’s Medicaid Expansion: An Unsolved Problem for Vulnerable Populations

**DOI:** 10.7759/cureus.50028

**Published:** 2023-12-06

**Authors:** Douglas P Nanu, Hardeep Tiwana, Michele M Carr

**Affiliations:** 1 Medical School, Washington State University, Spokane, USA; 2 Otolaryngology, Jacobs School of Medicine and Biomedical Sciences, University at Buffalo, Buffalo, USA

**Keywords:** affordable healthcare, medicaid expansion, affordable care act, south carolina, medicaid, healthcare policy

## Abstract

South Carolina is home to millions of residents and is renowned for its subtropical climate and beautiful beaches. Nevertheless, the state's healthcare system faces significant challenges, with a ranking in the bottom 50% of all states, a statistic that warrants serious attention. One of the most pressing healthcare issues in South Carolina is accessibility. The state currently has a higher percentage of its population that is medically uninsured compared to the national average in the United States (18% vs 14%, respectively). Consequently, a lower proportion of South Carolinians enjoy access to healthcare services when compared to residents of other states. Unfortunately, recent efforts to expand coverage for more of its residents via the Medicaid expansion by the Affordable Care Act (ACA) have been opposed by legislation. Lack of accessible healthcare is a significant issue in this state and the state legislature should increase access, especially to low-income vulnerable populations.

## Editorial

Global healthcare systems vary in how they provide healthcare to their citizens, and some manage to deliver effective care at a lower cost. Evaluating healthcare efficiency involves assessing several key factors, including access to care, the quality of care processes, administrative efficiency, equity, and overall health outcomes. Among eleven industrialized countries, including Australia, Canada, France, Germany, the Netherlands, New Zealand, Norway, Sweden, Switzerland, the United Kingdom, and the United States, the United States ranks 11th in terms of efficiency based on these criteria [1]. Compared to the United States, top-performing healthcare countries exhibit key qualities and services such as universal healthcare, significant investments in primary care systems, streamlined administrative processes, and greater investment in social services especially for children and working-age adults [1].

Regrettably, South Carolina's decision to reject Medicaid expansion does not contribute positively to enhancing healthcare efficiency within the United States. According to the *U.S. News & World Report*, South Carolina currently ranks 36th out of 50 states in terms of healthcare access, quality, and public health outcomes [2]. As per 2022 United States Census Bureau estimates, South Carolina is home to approximately 5,282,634 residents [3]. Among them, 1,350,213 million residents receive Medicaid benefits, but if the Medicaid expansion had been accepted, 351,000 residents would have also become eligible to receive Medicaid benefits [4]. Currently, to qualify for Medicaid in South Carolina, residents must meet one of the following criteria: 1) Being currently pregnant, 2) Being responsible for a child 18 years of age or younger, 3) Being blind, 4) Having a disability or having a family member in the household with a disability, or 5) Being 65 years of age or older [5]. In addition, a two-person household must have an income of no more than $19,720 to qualify, and no more than $5,140 for each additional person in the household [5]. For those who do not meet the criteria, their only option is to pay for insurance or to remain uninsured. Unfortunately, not all who do not qualify for Medicaid can afford insurance. People who comprise racial minorities are often the most affected by this issue. The U.S. Office of Management and Budget classifies minority racial and ethnic groups as American Indian or Alaska Native, Asian, Latino/Hispanic, Black or African American, and Native Hawaiian or Pacific Islander [6]. In South Carolina, the largest minority group is the Black or African American population, making up 26.3% of the total population [3]. These groups in South Carolina are selectively vulnerable to experiencing a gap in healthcare coverage. In South Carolina, 38% of Black people and 52% people of color are at risk of coverage gap due to having lower income than poverty guidelines to be eligible for Affordable Care Act (ACA) healthcare assistance [7]. However, these people are also not able to qualify for Medicaid because Medicaid expansion was not accepted by the state’s lawmakers in the argument that it would increase federal government spending [7,8]. The inability to address those who are uninsured health conditions promptly may lead to the development of chronic illnesses, which could have been preventable. This has the potential to eventually cause higher costs for the government in the long run compared to the amount required for early intervention and treatment.

Additionally, women of childbearing age are also at risk of losing benefits [7,9]. Women who are uninsured are at higher risk of having an unintended pregnancy due to an inability to access affordable reproductive health services, including contraception [9,10]. Medicaid expansion can reduce unwanted pregnancies by increasing access to reproductive health services [9,10]. During 2021, 13.5% of women in South Carolina of reproductive age were uninsured with 48.4% of mothers relying on Medicaid coverage [9]. Improving healthcare access and increasing favorable health outcomes is an important objective for the state. Currently, the state has a higher infant mortality rate than the national average (6.9 per 1,000 births vs. 6.0 per 1,000 births) [11]. Unfortunately, the infant mortality rate is even higher for Black women (12.7 per 1,000 births) [11]. Additionally, cancer-related deaths in this state are higher (179.0 per 100,000) than the national average (166.4 per 100,000) [11]. After accounting for differences in age makeup with other states, South Carolina had the 9th highest death rate in the United States [12].

Additionally, addressing healthcare disparities based on race and ethnicity factors is important. South Carolina has a high percentage of people of color with a lapse in coverage (52%) [7]. Additionally, Medicaid expansion would further provide coverage to over 120,000 children in states that did not pass the Medicaid expansion, South Carolina being one of them [7]. Expanding Medicaid in South Carolina not only improves healthcare access but also has the potential to decrease uncompensated care costs and save state resources. Currently, the American Rescue Plan offers South Carolina an extra $665 million to expand Medicaid, emphasizing the financial benefits of Medicaid expansion. With the expansion, the groups with the largest coverage gains would be the Black/African American populations [7]. A solution to improving healthcare in this state would be to extend Medicaid coverage, either by the ACA or another means. This could include creating a healthcare system that automatically provides insurance upon employment or a system such as the Canadian health system where citizens and permanent residents get health coverage.

Conclusion

Due to budget reasons, the legislature and the governor have opposed measures to expand access to healthcare for those residents who are in dire need of it. Medicaid expansion would increase access to affordable health insurance to uninsured minority groups and women of childbearing age. This decision disproportionately affects those with the greatest need for healthcare access, particularly those with low incomes, placing them at a distinct disadvantage in terms of receiving equitable care. If the state's lawmakers remain steadfast in their reluctance to expand healthcare access through Medicaid expansion, it is incumbent upon them to explore other possible avenues for expanding healthcare services, particularly for the vulnerable low-income populations that are disproportionately affected by this decision.
